# Non-Invasive Dynamic Reperfusion of Microvessels *In Vivo* Controlled by Optical Tweezers

**DOI:** 10.3389/fbioe.2022.952537

**Published:** 2022-07-14

**Authors:** Meng Shao, Min-Cheng Zhong, Zixin Wang, Zeyu Ke, Zhensheng Zhong, Jinhua Zhou

**Affiliations:** ^1^ Anhui Province Key Laboratory of Measuring Theory and Precision Instrument, School of Instrument Science and Optoelectronics Engineering, Hefei University of Technology, Hefei, China; ^2^ School of Biomedical Engineering, Anhui Medical University, Hefei, China

**Keywords:** optical tweezers, reperfusion, distributive shock, blood flow, *in vivo*

## Abstract

Distributive shock is considered to be a condition of microvascular hypoperfusion, which can be fatal in severe cases. However, traditional therapeutic methods to restore the macro blood flow are difficult to accurately control the blood perfusion of microvessels, and the currently developed manipulation techniques are inevitably incompatible with biological systems. In our approach, infrared optical tweezers are used to dynamically control the microvascular reperfusion within subdermal capillaries in the pinna of mice. Furthermore, we estimate the effect of different optical trap positions on reperfusion at branch and investigate the effect of the laser power on reperfusion. The results demonstrate the ability of optical tweezers to control microvascular reperfusion. This strategy allows near-noninvasive reperfusion of the microvascular hypoperfusion *in vivo*. Hence, our work is expected to provide unprecedented insights into the treatment of distributive shock.

## 1 Introduction

Distributive shock is a condition defined by the presence of the microvascular hypoperfusion despite the normalization of systemic and regional blood flow (Vincent and De Backer, 2013). In this case, fatal results will be caused when the oxygen delivery does not meet the metabolic needs of the tissue in vital organs (Pan et al., 2020). In order to keep the blood flowing in the microvessel and thus improve the oxygen delivery, several treatments such as fluid resuscitation, vasoactive drugs, positive inotropic drugs, obstruction relief, and mechanical assistance are commonly used (Legrand et al., 2018; Boros and Bauer, 2021; Jha et al., 2021; Jiang et al., 2021; Motwani and Saunders, 2021; Scheeren et al., 2021). In fact, these treatments with implementing methods aim to recover the macrocirculation function. However, an ideal target for resuscitation in clinics could be to improve microcirculation more promptly and accurately to avoid further organ damage. As a result, controlling microvascular reperfusion is an important strategy in the treatment of distributive shock. Although the development of several techniques such as magnetic (Galanzha et al., 2009), acoustic (Lenshof et al., 2009; Galanzha et al., 2016), and electrical devices (Wachter et al., 2011; Han et al., 2014) have reported positive effects in controlling the microvessel *in vivo* (Wang et al., 2014; Weijts et al., 2018), most of them required implantation of exogenous materials. To control blood microflow with high precision and in a non-invasive way, a biocompatible and single-cell-level strategy is highly desirable (Abdullah and Perez-Soler, 2012; Herbert et al., 2012; Sugden et al., 2017).

In this context, optical tweezers (Ashkin et al., 1986; Neuman and Block, 2004) have been used to manipulate and investigate microscopic particles for many years, and the ability to study living cells at the single-cell level have been proven (Zhang and Liu, 2008; Zhao et al., 2020; Vasse et al., 2021). Significant progress has been made in the manipulation of biological cells rotation with optical tweezers for orientation-based cell surgery (Xie et al., 2015a; Xie et al., 2015b; Xie et al., 2018a). In addition, study on the deformation of red blood cells (RBCs) by optical tweezers has long been a topic of real-life significance (Henon et al., 1999; Avsievich et al., 2020). Particularly, there have been developments toward optical trapping RBCs *in vivo* recently (Zhong et al., 2013a; Zhong et al., 2013b; Johansen et al., 2016; Horner et al., 2017; Zhong et al., 2017; Liu et al., 2020; Gao et al., 2021). Such as trap and manipulate RBCs within subdermal capillaries in living mice (Zhong et al., 2013a; Zhong et al., 2013b; Zhong et al., 2017). In addition, a recent report illustrates that optical tweezers were employed to manipulate, arrange, and rotate RBCs in the capillaries of zebrafish (Liu et al., 2020). However, optical trapping *in vivo* is still more challenging and complex due to scattering and power loss through biological tissue. To date, the experiments of optical micromanipulation *in vivo* has been applied to RBCs, injected nanoparticles, and macrophages (Johansen et al., 2016). Therefore, using optical tweezers, microvascular reperfusion *in vivo* may be carried out in a non-contact and non-invasive way.

In this study, a method for controlling dynamic reperfusion of mouse auricle microvessel using optical tweezers is introduced for the first time. We investigated the reperfusion ability by changing the position of optical trap and laser power when the reperfusion of microvascular branch was guided. The experimental results show that the control of microvascular reperfusion by optical tweezers at about 200∼550 mW of laser entering pupil is universal in the recovery of microvascular hypoperfusion. Moreover, a physical model was established to analyze reperfusion efficiency of the optical tweezers with the laser power. According to our analysis, as the laser power increases, the reperfusion efficiency increases at first and then decreases. The implementation of this technique is almost non-invasive in controlling dynamic reperfusion of microvessel *in vivo*, which has not yet been achieved by any other techniques, thereby gaining novel insights into the treatment involved in distributive shock.

## 2 Materials and Methods

### 2.1 Optical Tweezer Setup

**FIGURE 1:**
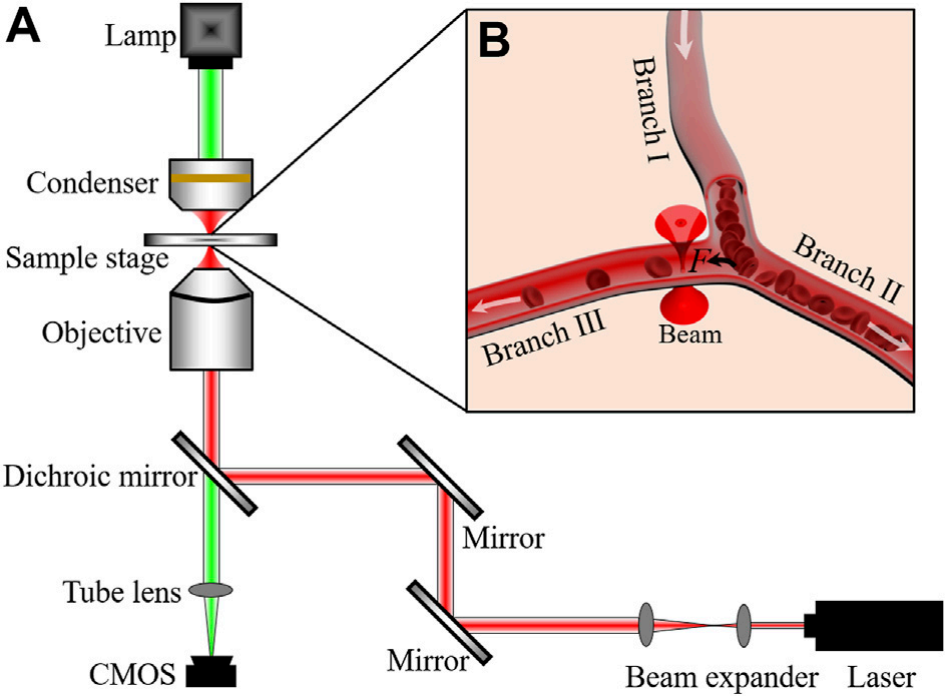
**(A)** Optical tweezers setup. **(B)** Schematic illustration of blood flow controlled by optical trap. White arrows indicate the directions of blood flows. ***F*** is the optical trapping force.

Our optical tweezers setup is based on an Olympus IX73 inverted microscope, as shown in Figure 1A. A 1064-nm laser (Amonics, Hong Kong, AFL-1064-37-R-CL, cw) was used as the trapping laser source. The laser beam was expanded to fulfill the pupil of objective (8 mm) with the beam expander. For better trapping and imaging of RBCs in the microvessel of the pinna of mice (Zhong et al., 2013a), a water immersion objective (LUMFLN, × 60, numerical aperture 1.20, Olympus, Japan) was also used here. A CMOS camera (MindVision, China, MV-SUA231GM-T, 20 frames per second) was used to connect to the computer for the real-time image acquisition and video recording of the intravital manipulation. The laser power was measured at the pupil of the objective.

### 2.2 Mice Preparation

Kunming mice (8-week-old, 30 ± 2 g) were used in the experiments. They were purchased from the Experimental Animal Center of Anhui Medical University. To ensure that the mice keeps alive and immobilized during the whole operation process, 10% chloral hydrate (7 μl·g^-1^) was injected *via* the abdominal cavity to anesthetise them. The hair on the pinna of the mice was removed by cream and then glycerin was smeared on the pinna for avoid drying out the skin. During the experiments, the optical trap (red cone beam in Figure 1B) was located at a branch of the microvessel. When the optical trap applied an attractive force on an RBC passing the junction of microvessel, the cell will be guided to the Branch Ⅲ and added the number of RBCs in Branch Ⅲ, as schematized in Figure 1B. The study was approved by the Ethical Committee of the Hefei University of Technology.

## 3 Results and Discussion

### 3.1 Optical Trap–Controlled Reperfusion of a Microvessel

**FIGURE 2:**
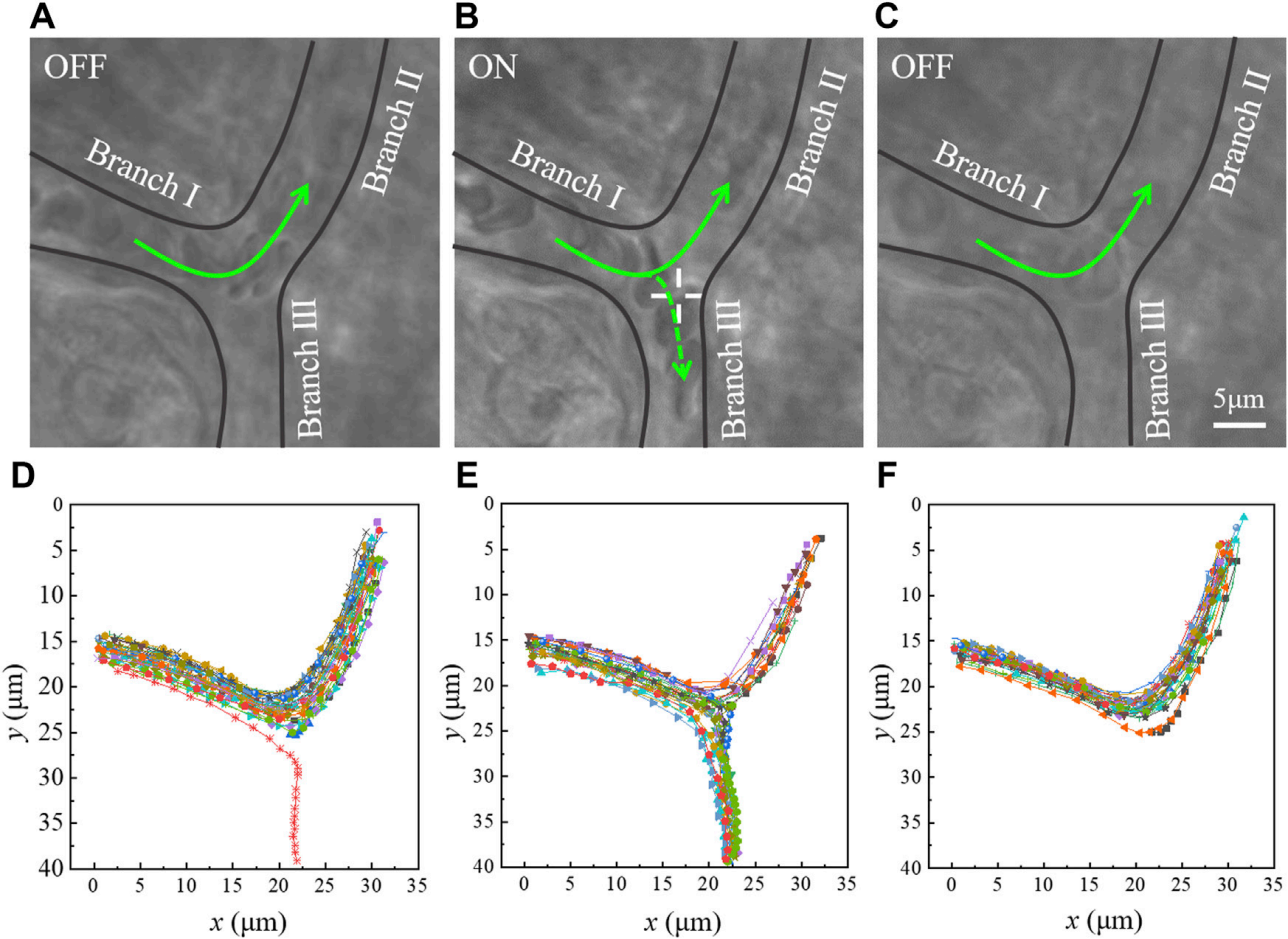
Dynamic reperfusion of the microvascular blood flow by optical tweezers. The laser is **(A)** off (Supplementary Video S1), **(B)** on (Supplementary Video S1), and **(C)** off (Supplementary Video S1). ‘+’ indicates the optical trap. **(D**–**F)** Path tracing of the RBCs. The points record the position coordinates of RBCs in each frame of the images, and each curve represents the flowing path of an RBC.

The optical tweezers can only manipulate the RBCs in depth smaller than 100 μm. Here, we have demonstrated the insufficient microvascular perfusion in a branching microvessel, which was with ∼40 μm depth beneath the surface of mice pinna skin, as in Figure 2. As shown in Figure 2A and Supplementary Video S1, the RBCs flowed from Branch Ⅰ into Branch Ⅱ and Branch Ⅲ in a certain proportion, but there was not enough or even no blood flowing to Branch Ⅲ. This phenomenon was very common in the pinna of anesthetized mice, which might due to the hypothermia of the anesthetized mice. The paths of all RBCs within 4 s were traced using ImageJ software. The tracking results were presented in Figure 2D, and only one RBC entered Branch Ⅲ in 4 s. When the optical trap worked on the Branch Ⅲ, the blood flow was redistributed. One example was demonstrated in Figure 2B and Supplementary Video S2. The laser power was 221 mW. It can be observed that some RBCs were directed into Branch Ⅲ, and the tracing of RBCs was shown in Figure 2E. As the optical trap was turned off again, the phenomenon of insufficient RBC perfusion in Branch Ⅲ appeared again, as shown in Figure 2C (Supplementary Video S3) and Figure 2F. The experiment results showed that the optical trap can apply force on the RBCs flowing in the microvessels, which would change the blood distribution inside the microvascular branches.

### 3.2 Universal Applicability of Optical Trap–Controlled Reperfusion

**FIGURE 3:**
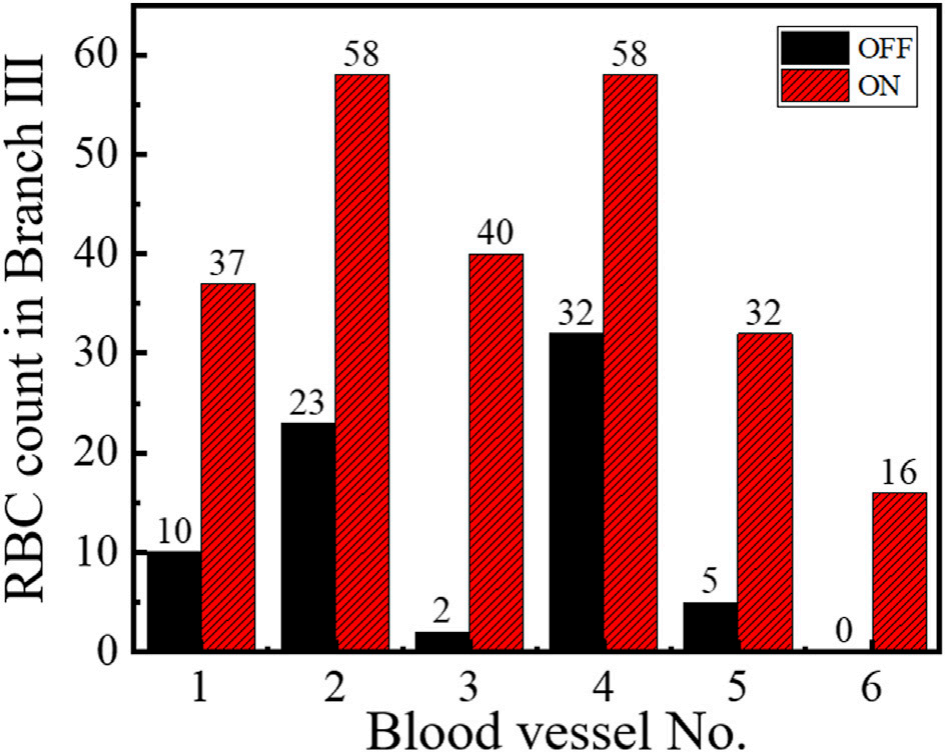
RBCs count of reperfusion in six different branching microvessels within 10 s.

To check the universal applicability of microvascular reperfusion by optical tweezers, we conducted a series of experiments. Figure 3 shows the RBC counts of the six groups of branch vessels under the optimal condition of optical tweezers, respectively. The recording time for each group was 10 s, and the RBCs were counted at the microvascular Branch Ⅲ. The red bar represented the optical trap was working, and the black bar represented the optical trap was not working. The experimental results in Figure 3 showed that optical tweezers can increase the perfusion of RBCs in different microvessels. For the microvessels 1–5 with low blood flow perfused, the average rate of improvement was 588%. The maximum number of microvascular reperfusion cells could increase by 1900% for the microvessel 3 controlled by optical tweezers. The analysis of experimental results showed that the optical tweezers could control their reperfusion for the low perfused microvessels. The efficiency of optical tweezers–controlled reperfusion was significant, especially in the case of severe microvascular hypoperfusion.

### 3.3 Effect of Optical Trap Position on Reperfusion Efficiency

In the reperfusion experiments at different positions, an effective redirection of RBCs was required. In microfluidic channels, the efficiencies of cell manipulation and sorting can be enhanced by moving the position of optical trap (Wang et al., 2011; Landenberger et al., 2012; Zhou et al., 2021). Here, the suitable position of the optical trap should be obtained for redirection of RBCs in the microvessels *in vivo*.

**FIGURE 4:**
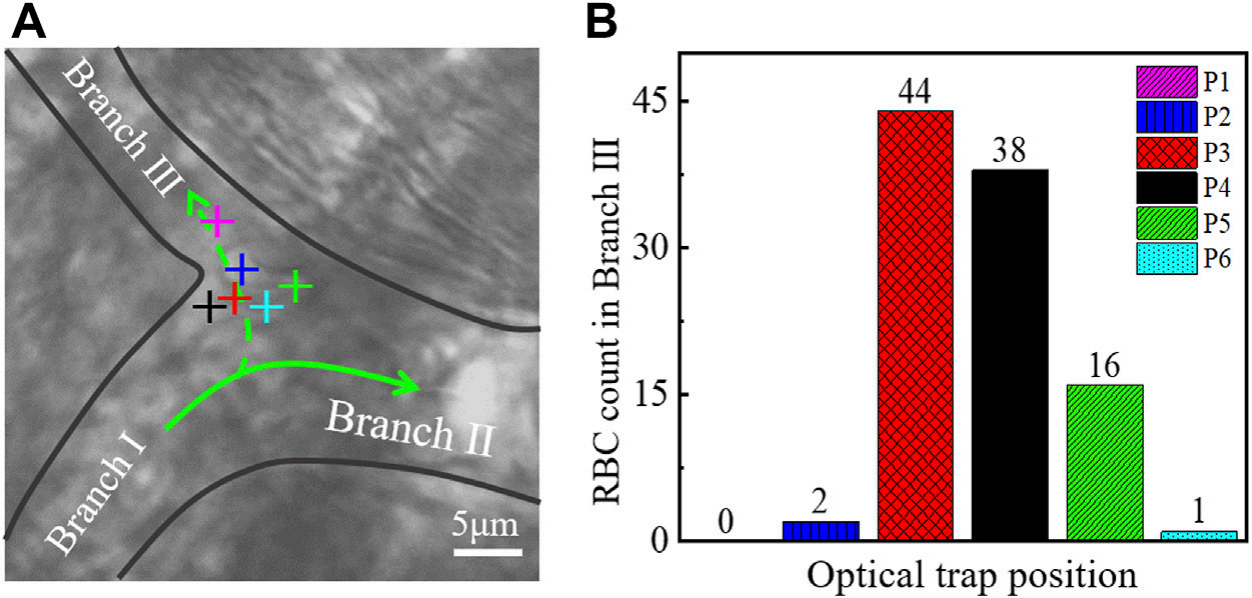
**(A)** Optical trap was located in different positions in a microvessel. Different colors of ‘+’ represent different positions of the optical trap. **(B)** Relationship between the position of the optical trap and the amount of redirection RBCs. The color of the histogram corresponds to the color of the optical trap in **(A)**. ‘+’ indicates the optical trap.

An experiment was conducted to determine the relationship between optical trap position and reperfusion efficiency as shown in Figure 4A. We selected six typical positions at the branch of vessels for optical trap working, as marked with ‘+’ in Figure 4A. Then we recorded the blood flow with 30 s videos for each working location, and the erythrocytes in Branch Ⅲ were counted. The results were indicated in Figure 4B, the microvascular reperfusion was obviously better when the optical trap position was located at P3 (red) and P4 (black). The reason behind such better performance of the microvascular reperfusion might be that the optical trap was redirecting the RBCs rather than trapping them. Fortunately, the optical trap can change the flowing direction of RBCs and then attracted the RBCs into the Branch Ⅲ. Therefore, the optimal optical trap position for the reperfusion can be determined roughly. At P3 and P4 in Figure 4A, the redirected RBCs were trapped and flowed into the Branch Ⅲ one by one.

When the optical trap was located at P3 (red), P4 (black), and P5 (green), some RBCs flowing to Branch Ⅱ initially were within the action range of the optical trap. These RBCs can be attracted and flowed to the Branch Ⅲ eventually. The optical trap has reperfusion ability. When the optical trap was located at P1 (purple) and P2 (blue) as in Figure 4A, the most of RBCs flowing to Branch Ⅱ were not in the action range of optical trap, so the numbers of reperfusion RBCs were poor. When the optical trap was located at P6 (cyan) as in Figure 4A, the optical trap has no reperfusion capability because the optical trapping force cannot overcome the dragged force from the blood flow of Branch Ⅱ, and the RBCs cannot redirect and flow to Branch Ⅲ.

Since the geometry of each branch *in vivo* is different, it is difficult to determine a unified standard to apply to all branches when the relationship between the optical trap position and the reperfusion efficiency was investigated. The results shown in Figure 4 suggest that the optical trap position is one of the important factors affecting the efficiency of optical tweezers to control microvascular reperfusion. Combined with experiments, however, the possible optimal location for reperfusion can still be given. We believe that the optimal trap location is more likely to be near the branch entrance. Microvascular reperfusion could be achieved when the optical trap location within the range of is ∼2 μm near the branch entrance. In fact, this range is easily identified.

### 3.4 Effect of Laser Power on Reperfusion Efficiency

**FIGURE 5:**
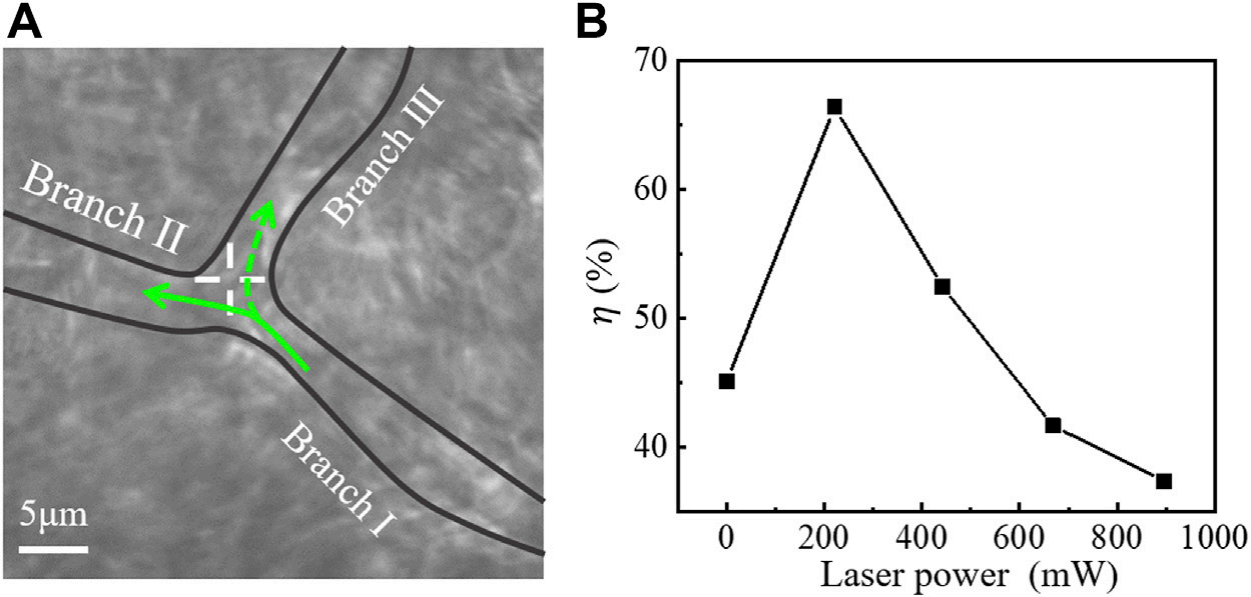
**(A)** Optical tweezers control of blood flow in a branching microvessel. ‘+’ indicates the optical trap. **(B)** Relationship between laser power and reperfusion efficiency (*η*).

To investigate the effect of laser power on reperfusion, the optical trap position was fixed at the optimal location, as shown in Figure 5A, and the laser power was gradually increased for reperfusion experiment. In this experiment, the 30-s videos were recorded at each power. The reperfusion efficiency *η* is given as *η*=(*q*_Ⅲ_/*q*_Ⅰ_)×100%, where *q*_Ⅰ_ and *q*_Ⅲ_ represented the RBC amounts flowed into Branch Ⅰ and Branch Ⅲ, respectively. Figure 5B showed the reperfusion efficiency with increasing laser power. The results showed that optical trap cannot control the reperfusion of RBCs when the power of laser entering pupil was less than 200 mW for this microvessel. When the laser power was about 200∼550 mW, reperfusion efficiency decreased with increasing laser power. When the laser power was greater than 550 mW, the RBCs were trapped at the entrance of Branch Ⅲ and decreased the blood flow of Branch Ⅲ.

The experimental results reported in Figure 5 indicate that an appropriate laser power was crucial in determining the ability of blood reperfusion. For *in vivo* trapping, the maximum tested power was almost up to 1000 mW, the heat-induced thermal damage should be discussed. During the reperfusion controlled by optical tweezers, there was no observable damage to microvessels or cells. And no burning spots were observed on the pinna of the mice for several weeks after the experiments. Studies have shown that under the same trapping conditions, Chinese hamster ovary cells exhibit an average temperature rise of nearly 1.15 ± 0.25 °C/100 mW (Liu et al., 1995). This thermal damage, which can be almost ignored, seems to be explained by the weaker absorption of organisms at 1064 nm wavelength relative to visible light (Liu et al., 1995; Liu et al., 1996; Neuman et al., 1999; Peterman et al., 2003). In addition, RBCs with high thermal conductivity can carry away some of the heat induced by the absorption through the fast blood flow, and this also prevents local thermal accumulation (Gao et al., 2021). In Section 3.5, we will analyze the relationship between reperfusion efficiency and the laser power.

### 3.5 Physical Model

**FIGURE 6:**
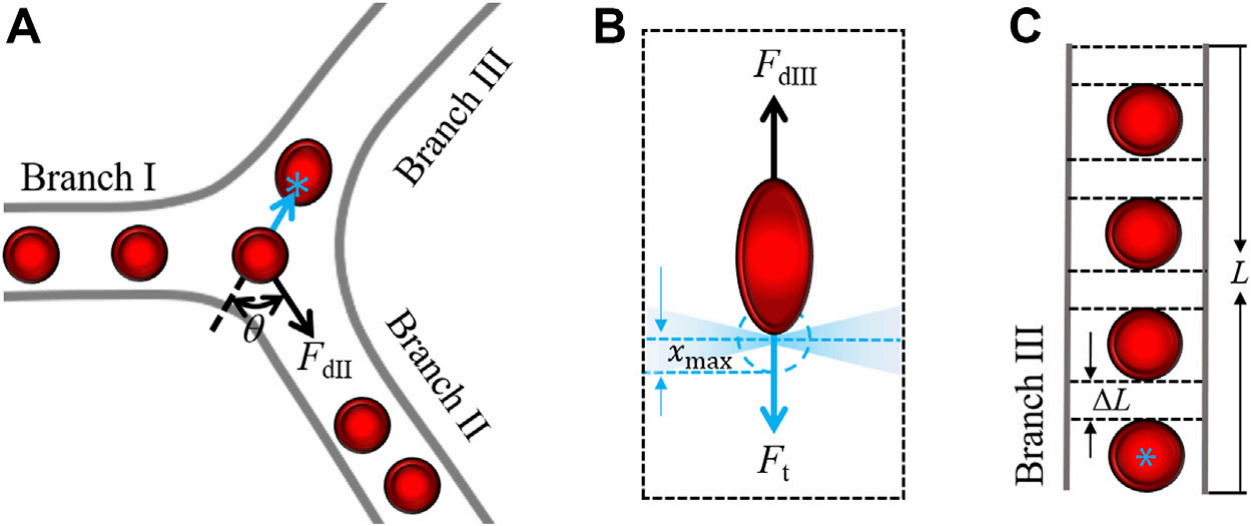
Theoretical analysis model. **(A)** RBC is redirected and flows to Branch Ⅲ under the action of optical trap. ‘*’ indicates the optical trap. **(B)** Forces on the RBC being dragged by the blood flow. *x*_max_, the maximum action range of trapping force; **(C)** RBCs in Branch Ⅲ. *F*_t_, optical trapping force; *F*_dⅡ_ and *F*_dⅢ_ represents drag force from blood flow in Branch Ⅱ and Branch Ⅲ, respectively; and Δ*L*, increased length without RBC in the microvessel during the staying time *t*.

The microvascular reperfusion efficiency is correlated with the optical trap force, which is proportional to the laser power. To explain reperfusion efficiency of the optical tweezers with the laser power, a physical model was established. The forces on an RBC passing junction is described in Figure 6A, the cell will flow into Branch Ⅱ without the optical trap working. We assumed that blood flows are steady flows in all branches. The RBC experiences a drag force *F*_dⅡ_ from blood flow of the Branch Ⅱ. When the laser power was larger than a critical trapping power *P*_c_, the optical trapping force *F*_t_ can overcome the component of drag force *F*_dⅡ_cos*θ*, and an RBC originally flowing from Branch Ⅰ to Branch Ⅱ can be redirected to Branch Ⅲ, as in Figure 6A.

From the experimental videos (Supplementary Video S4), the redirected RBCs were stretched in the trap by the drag force *F*_dⅢ_ from the blood flow of Branch Ⅲ. The forces on the trapped RBC are described in Figure 6B. When the RBC passes through the trap, the cell will be trapped at the position where the *F*_dⅢ_ and *F*_t_ are balanced. The *F*_t_ acts on a part of the RBC actually. Here, the effective part of the RBC is described with stretch ratio (*λ*) as in Figure 6B. The stretch ratio *λ* is defined as the ratio of the length of an RBC stretched to its diameter. Before the RBC is stretched, initial stretch ratio is set as *λ*_0_. With the increase of the optical trapping force, the stretching ratio of RBC increases. The optical trapping force is proportional to the stretch ratio of trapped object (Rohrbach, 2005). When the RBC is stretched to long enough, the *F*_t_ will be smaller than *F*_dⅢ_, and the RBC will escape from the trap. The escaping RBC enters Branch Ⅲ and increases the number of RBCs in Branch Ⅲ. However, the RBC cannot be stretched to infinity, so there is a maximum stretching ratio (*λ*_max_). When the *F*_t_ increases with increasing laser power to a certain value *P*_m_, the RBC will be stretched to the maximum stretch ratio, the *F*_t_ (*λ*_max_) can overcome the drag force *F*_dⅢ_. The RBC will be trapped and clog the entrance of Branch Ⅲ, the number of RBCs in Branch Ⅲ will be smaller than that of before the optical trap working. The relation between the laser power *P* and the trapping force *F*_t_ is summed as follows:

$$\{\begin{array}{c} F_{trap}<F_{dⅡ}cos\theta ,\;\;\;\;\;\;\;\;\;P<P_{c},\\ F_{dⅡ}\cos\theta \leq F_{t}\left(\lambda_{0}\right)\&F_{t}\left(\lambda_{\max}\right)\leq F_{dⅢ},\;\;\;\;\;P_{c}\leq P\leq P_{m}\\ F_{t}\left(\lambda_{\max}\right)>F_{dⅢ},\;\;\;\;\;\;\;\;\;\;\;\;\;\;\;\;\;\;\;\;P>P_{m}, \end{array}.$$ (1)

In Equation 1, we describe the different relationship between the optical trapping force and the blood flow drag force on RBC caused by the increase of laser power. The further analysis is the effect of optical trapping force on microvascular reperfusion efficiency with the increase of laser power. Here, the time *t* spent in the range of optical trap for each RBC was used to reflect the efficiency of reperfusion. The cell flowing to Branch Ⅱ initially is trapped at the entrance of Branch Ⅲ due to the action of optical trapping force. Then, the RBCs in the optical trap are stretched under the drag force of blood flow, and the relationship between the maximum stretch ratio *λ*_max_ and time *t* can be expressed as follows (Hochmuth et al., 1979; Lim et al., 2004):

$$\frac{\left(\lambda_{0}^{2}-1\right)\left(\lambda_{\max}^{2}+1\right)}{\left(\lambda_{0}^{2}+1\right)\left(\lambda_{\max}^{2}-1\right)}=\exp\left(-\frac{t}{t_{c}}\right).$$ (2)

When the RBC is stretched to the maximum stretched length, it escapes from the optical trap. *t*_c_ is the characteristic time (Hochmuth et al., 1979; Lim et al., 2004). If the cell can escape from the optical trap, the cell receives the maximum optical trapping force when it is at *x*_max_ from the optical trap center. In fact, *x*_max_ may increase slightly as the laser power increases. In this case, the stretch ratio of cell is the maximum, as in Figure 6B. The maximum stretch ratio *λ*_max_ of an RBC can be estimated by (Evans, 1983; Parker and Winlove, 1999; Sleep et al., 1999)

$$\lambda_{\max}=\frac{kx_{\max}}{\sqrt[{3}]{125BrH^{2}}},$$ (3)

where *H* and *B* represent Shear modulus and bending modulus of cell membrane, respectively, *r* is the initial radius of the RBC, and *k* represents the optical trap stiffness. Therefore, the time for cell staying in the optical trap *t* can be described as follows:

$$t=-t_{c}ln\left(\frac{\left(\lambda_{0}^{2}-1\right)\left(k^{2}x_{\max}^{2}+25\sqrt[{3}]{B^{2}r^{2}H^{4}}\right)}{\left(\lambda_{0}^{2}+1\right)\left(k^{2}x_{\max}^{2}-25\sqrt[{3}]{B^{2}r^{2}H^{4}}\right)}\right).$$ (4)

When the laser power increases, *k* and *t* increases, as shown in Eqn. 4. In other words, with the increase of laser power, the optical trapping force acting on RBC will be increased, thus increasing the residence time of RBC at the branch. For simplicity, we assume the RBCs flow into the Branch Ⅲ one by one. The number of RBCs in the Branch Ⅲ before optical trap working is represented as *q*_0_, the number of reperfused RBCs is set as Δ*q* when the optical trap can work at the laser power *P*_c_, and the blood flow velocity *v*_*f*_ is assumed as a constant. For the Branch Ⅲ with length *L*, the ‘rescaled’ diameter of RBC *d* can be obtained by *d* = *L*/(*q*_0_+Δ*q*). When the laser power is larger than the *P*_c_, the RBC stays in the optical trap within the time *t*, and there is an increasing RBC unfilled length Δ*L* during the time *t*, as in Figure 6C. The numbers of reperfusion RBCs can be expressed as follows:

$$\Delta q=\frac{L}{d+\Delta L}-q_{0}=\frac{L}{d+v_{f}t}-q_{0}.$$ (5)

From Eqn. 4, the staying time *t* increases with increasing laser power and optical stiffness. Therefore, the number of reperfusion RBC Δ*q* decreases with increasing laser power. When the laser power increases to larger than *P*_m_, the cell cannot escape from the optical trap. The trapped RBCs will clog the entrance of Branch Ⅲ, and stop the other flowing RBCs into Branch Ⅲ. According to the above analysis, we can express the effect of optical trap on RBC reperfusion controlling as follows:

$$\Delta q\{\begin{array}{c} =0,\;\;\;\;\;\;\;\;\;\;\;\;\;\;\;\;\;\;\;\;\;\;\;\;\;\;\;\;\;\;\;\;\;\;\;\;\;\;P<P_{c}\;,\\ =\frac{L}{d+\Delta L}-q_{0}=\frac{L}{d+v_{f}t}-q_{0},\;\;\;\;\;\;P_{c}\leq P\leq P_{m},\\ <0,\;\;\;\;\;\;\;\;\;\;\;\;\;\;\;\;\;\;\;\;\;\;\;\;\;\;\;\;\;\;\;\;\;\;\;\;P>P_{m}. \end{array}$$ (6)

According to the Eqn. 6, the results in Figure 5B can be explained qualitatively. When the laser power was less than 200 mW, the optical trapping force was smaller than the drag force of blood flow, the all RBCs were dragged into the Branch Ⅱ, and the reperfusion of RBCs to Branch Ⅲ cannot be controlled. When the laser power was 200∼550 mW, the optical trapping force can guide the reperfusion of RBCs to Branch Ⅲ. However, when the power was greater than 550 mW, RBCs were trapped in the optical trap at the entrance of Branch Ⅲ, resulting in microvascular obstruction. The cells initially flowing to Branch Ⅲ were also inhibited, which worsened the microvascular ischemia of Branch Ⅲ.

Since the complexity of the environment *in vivo*, the blood pressure of different microvessels in pinna of mice may be slightly different. This leads to differences in the optimal laser power for controlling reperfusion. However, the reperfusion experiments by optical tweezers in this manuscript were all carried out at 200∼550 mW of laser entering pupil. Only one data is shown in Figure 4, illustrating the effect of laser power on reperfusion, but visualizations in Figure 3 show the universal applicability of reperfusion.

In the experiment, the optical trapping force decreased with the increase of the depth of the reperfusion microvessels. This is the result of laser energy loss due to the strong scattering characteristic of biological tissue. With the depth of the optical trap inside the body increasing, the loss of laser energy will be increased. Moreover, the trap stiffness decreases with the increase of laser optical trap depth. The solution of these two problems requires increasing laser power, thus affecting the optimal laser power of reperfusion. Therefore, the optimal laser power for optimal reperfusion is within a range.

To improve the trap stiffness of the optical trap position, increasing laser power is an effective strategy. However, this may cause photodamage to blood vessels and RBCs. Therefore, methods to reduce light damage need to be used. Recently, some practical progress has been made toward decreasing photodamage in optical trapping systems. Azimuthally polarized beams achieve higher axial trapping efficiency and lower photodamage than linearly polarized Gaussian beams when used for optical trapping of individual RBCs (Yu et al., 2020). Moreover, many indirect-based cell manipulation strategies have been developed to significantly avoid photodamage to the target cell (Chowdhury et al., 2013a; Xie et al., 2018b; Stoev et al., 2021; Chen et al., 2022). Previous reports suggesting that the photodamage can be totally eliminated for pushing-based cell manipulation, as opposed to occurrence rates of 67 and 33% for direct trapping and tool attachment or gripping, respectively, (Chowdhury et al., 2013b; Banerjee et al., 2014). Therefore, reducing the contact between the laser and the target cell is an effective strategy to avoid photodamage in optical trap manipulation using near-infrared laser. We consider optical tweezers combined with these indirect-based manipulation methods could help achieving reperfusion of microvessels with deeper.

## 4 Conclusion

In conclusion, we have derived an efficient method of microvascular reperfusion *in vivo*, and it is the first time to reperfusion the microvascular hypoperfusion in the pinna of mice. The effect of the position of the optical trap center and the laser power on the reperfusion was studied experimentally for carrying out the experiment of reperfusion under suitable optical trap position and laser power. The results showed that microvascular reperfusion could be achieved by optical tweezers near the branch entrance of microvascular hypoperfusion with 200∼550 mW of laser entering pupil. In addition, a series of experiments have demonstrated the universal applicability of microvascular reperfusion by optical tweezers.

As far as we know, the current treatment for distributive shock ensures blood perfusion of macrocirculation. Precise treatment of the microvascular hypoperfusion is difficult (Vincent and De Backer, 2013; Pan et al., 2020). The development of various techniques has provided new ideas for the treatment of the microvascular hypoperfusion (Galanzha et al., 2009; Lenshof et al., 2009; Galanzha et al., 2016). In this study, optical tweezers were used to control microvascular reperfusion at branch. Experimental results on the pinna of mice show that the method is effective in the treatment of the microvascular hypoperfusion. The implementation of this technique is almost non-invasive in controlling dynamic reperfusion of microvessels *in vivo*, which has not yet been achieved by any other techniques. However, the treatment of distributive shock with this technique still faces some challenges. One is that traditional Gaussian optical tweezers cannot form an effective optical trap in deep tissue due to the strong scattering characteristics of biological tissue, and the other is the lack of real-time and clear imaging methods in deep tissue. This is why we are working in mice pinna.

Moreover, experimental results indicate that optical trapping force should be maintained at the branch in order to treat microvascular hypoperfusion. The method we propose will be a pertinent treatment of hypoperfusion if the optical trapping force remains at the branch for a short time, allowing continuous reperfusion of microvessels. We will seriously discuss and pay attention to this issue in the following research.

As a basic study, however, optical tweezers can be used as a new method to control microvascular reperfusion *in vivo*, and this non-invasive and precise method of microvascular reperfusion is expected to provide new insights into the treatment of distributive shock.

## Data Availability

The original contributions presented in the study are included in the article/Supplementary Material; further inquiries can be directed to the corresponding authors.
